# Emphysematous changes as red flag signs preceding rapidly progressive infectious aortic disease: two case reports

**DOI:** 10.1186/s12872-023-03619-8

**Published:** 2023-11-21

**Authors:** Yusuke Higuchi, Tetsuya Nomura, Shiori Yoshida, Michitaka Kitamura, Kenshi Ono, Keisuke Shoji, Naotoshi Wada, Natsuya Keira, Tetsuya Tatsumi

**Affiliations:** Department of Cardiovascular Medicine, Kyoto Chubu Medical Center, 25, Yagi-Ueno, Yagi-cho, Nantan City, Japan

**Keywords:** Case report, Infectious aortic disease, Emphysematous changes, Red flag sign

## Abstract

**Background:**

Infectious aortic disease is a rare and fatal disease, that requires the appropriate intervention. An accurate diagnosis should be promptly established. However, this is difficult because the clinical manifestations of this disease vary and are non-specific.

**Case presentation:**

(CASE 1) An 87-year-old male, presenting with generalized malaise and weight loss, was admitted for further examination. A chest computed tomography (CT) showed mediastinal emphysema. Empirical intravenous antibiotics were administered to address the non-specific infectious findings in the laboratory data. The treatment was effective, and the patient fully recovered. However, he was in shock due to aortic rupture and marked pseudo aneurysmal formation around the aortic arch day 25 of hospitalization. An emergency total aortic arch replacement was performed, and the patient was discharged. (CASE 2) An 82-year-old male who had undergone Y-graft replacement in the abdominal aorta 15 years previously was admitted due to general malaise and anorexia. Abdominal CT revealed emphysematous changes adjacent to the abdominal aorta. The patient responded favorably to empirical treatment with intravenous antibiotics and was discharged 19 days after admission. Four days after discharge, the patient went into cardiac arrest after an episode of hematemesis. Abdominal CT revealed an enlarged stomach and duodenum, filled with massive high-density contents proximal to the abdominal aorta. He died of hemorrhagic shock despite cardiopulmonary resuscitation.

**Conclusions:**

Although emphysematous changes are rare, they are red flag signs during the early stage of infectious aortic disease. Thus, physicians should remain vigilant for this kind of critical sign.

## Background

Infectious aortic aneurysm constitutes 2.6% of aortic aneurysms [1]. It is a rare and fatal disease, that can be treated appropriately. The successful treatment of infectious aortic aneurysms depends on a prompt and accurate diagnosis, followed by efficient antibiotics and timely surgery. However, this is difficult because the clinical manifestations of this disease are non-specific, depending on the degree of infection and subsequent aneurysmal formation [2].

## Case presentation

### (CASE 1)

**Fig. 1:**
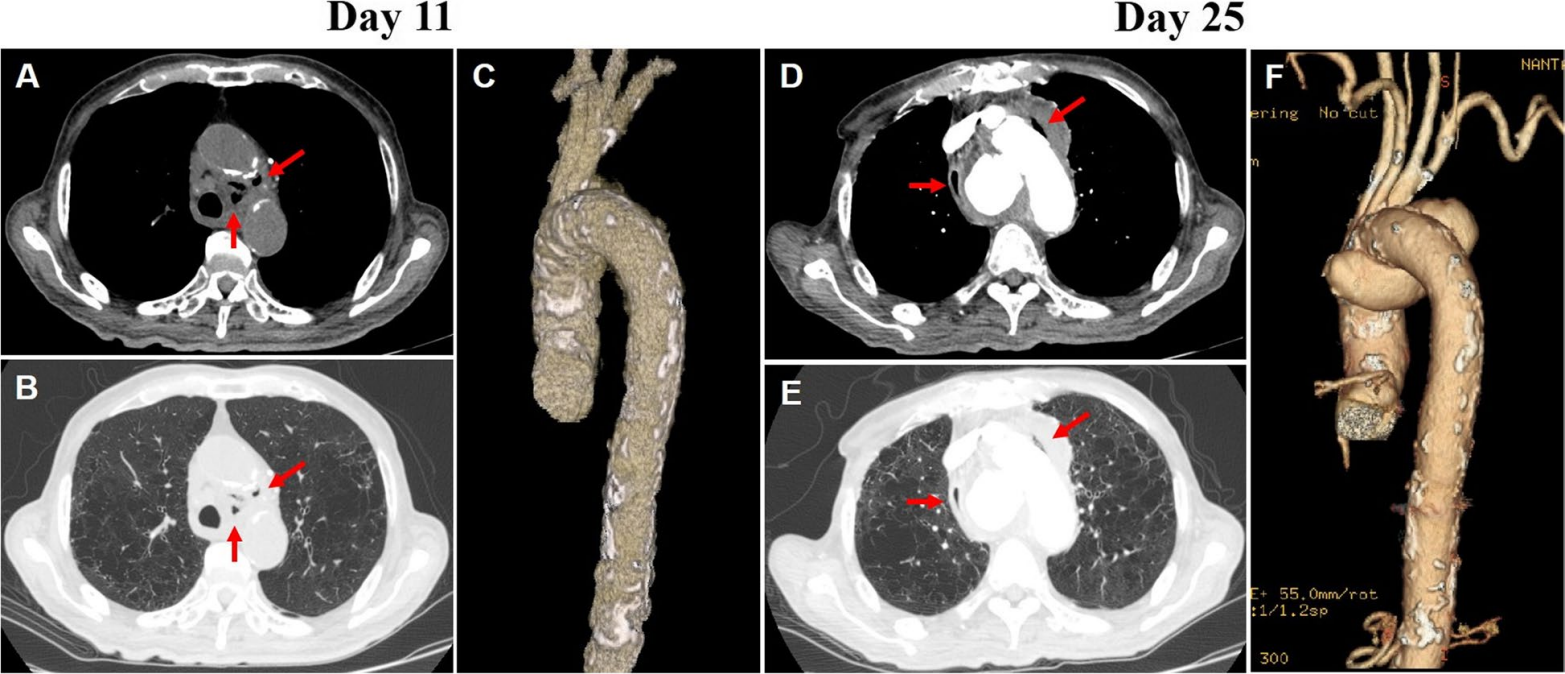
**a**, **b** A chest computed tomography (CT) showing mediastinal emphysema (Arrows). **c** The thoracic aorta exhibited some atheromatous changes but no aneurysmal formation. **d**, **e** A contrast-enhanced chest CT showed peri-aortic emphysema (Arrows). **f** Three-dimensional CT angiography demonstrated marked pseudo aneurysmal formation around the aortic arch

An 87-year-old male, presenting with generalized malaise and weight loss of 10 kg over two months, was admitted to our hospital for further examination about his clinical conditions and improving his nutritional status. The patient had diabetes mellitus (DM) and hypertension. His DM condition was well controlled that the latest HbA1c value was 5.5%. On admission, he had a blood pressure of 146/84 mmHg and a pulse rate of 74 bpm. His body temperature was 36.0℃, and peripheral pulse was normally palpable. No abnormal findings were observed on physical examination. Abdominal computed tomography (CT) scan showed chronic pancreatitis. Laboratory studies demonstrated several inflammatory findings, such as C-reactive protein (CRP) of 9.3 mg/dL and white blood cell (WBC) of 9,480 /mm^3^. Eleven days after admission, the patient remained asymptomatic, but he had a decreased hemoglobin of 8.1 g/dL, elevated CRP of 14.1 mg/dL, and elevated WBC of 14,100 /mm^3^. Chest CT was performed to rule out aspiration pneumonia, and happened to reveal mediastinal emphysema. The wall structure of the esophagus was clearly maintained, and there seemed no involvement between esophagus and mediastinal emphysema (Fig. 1a, b Arrows). The thoracic aorta exhibited some atheromatous changes, but no aneurysmal formation was observed (Fig. 1c). After collecting two blood culture specimens, empirical intravenous antibiotics (Cefpirome 2 g/day) were administered. The patient responded favorably, and he regained his appetite on day 14. His laboratory results were also improved (CRP 5.7 mg/dL, WBC 7,340 /mm^3^). Thereafter, he fully recovered, and antibiotics administration was stopped on day 22.

On admission day 25, the patient complained of severe chest pain. He developed sudden respiratory failure and shock. Laboratory studies showed several inflammatory findings, such as CRP of 9.3 mg/dL and WBC of 9,480 /mm^3^. D-dimer was measured 3.6 µg/mL. Bed side transthoracic echocardiography showed no remarkable findings of wall motion abnormality and valvular disfunction. Contrast-enhanced chest CT showed peri-aortic emphysema (Fig. 1d, e Arrows) Moreover, three-dimensional CT angiography demonstrated aortic rupture and marked pseudo aneurysmal formation around the aortic arch (Fig. 1f). The patient was referred to the unit of cardiovascular surgery in other hospital. Emergency surgery was performed, and showed ruptured aortic arch and pseudo aneurysm was filled with hematoma. Abscess formation was also found around the aneurysm. After removing infectious lesions, total arch replacement was performed. *Klebsiella pneumoniae* was detected in both sputum and blood cultures. The species was susceptible to cephem antibiotic. Based on this, the appropriate antibiotics were administered post operative days. The patient had an uneventful hospital course and was eventually discharged.

### (CASE 2)

**Fig. 2:**
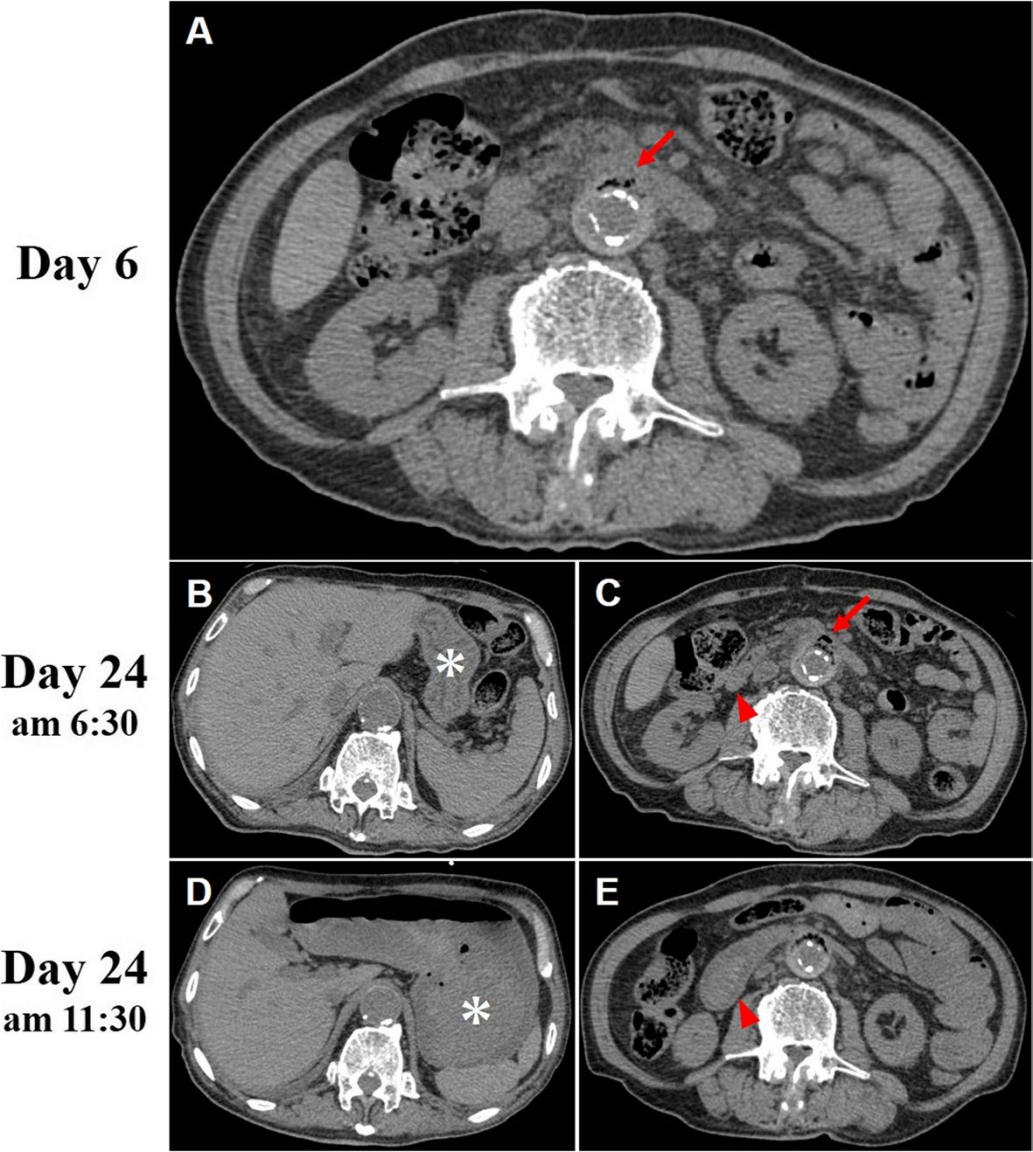
**a** An abdominal CT showed emphysematous changes adjacent to the abdominal aorta (Arrow). **b**, **c** Plain CT images on re-admission (asterisk; stomach, Arrowhead; duodenum, Arrow; emphysematous changes). No remarkable change compared to the CT findings of Day 6. **d**, **e** An abdominal CT revealed an enlarged stomach (Fig. **d** Asterisk) and duodenum (Fig. **e** Arrowhead), filled with high-density massive contents proximal to the area adjacent to the abdominal aorta

An 82-year-old male who had comorbidities of hypertension and dyslipidemia was admitted to our hospital for general malaise and anorexia. About 15 years ago, the patient underwent Y-graft replacement in the abdominal aorta for an abdominal aortic aneurysm. On admission, he had a blood pressure of 132/76 mmHg and a pulse rate of 80 bpm. The body temperature was 36.7℃, and laboratory data showed several inflammatory findings, such as CRP of 3.9 mg/dL and WBC of 8,320 /mm^3^, and deteriorated renal function (Cre 2.91 mg/dL, eGFR 17.1 mL/min/1.73m^2^). Abdominal CT on admission day 6 revealed emphysematous changes, adjacent to the abdominal aorta (Fig. 2a Arrow), but the patient reported no abdominal symptoms. During this time, the focus of the inflammatory findings was not identified. However, the empirical intravenous antibiotics (Ceftriaxone 2 g/day) were still administered until the discharge. The patient gradually recovered, and the laboratory results normalized. The patient was discharged 19 days after admission.

Four days after his discharge, he returned to our emergency room for epigastric pain, occurring in the morning. Abdominal CT showed no remarkable change compared with the findings 18 days earlier. The emphysematous changes adjacent to the abdominal aorta was also detected (Fig. 2b, c Arrow). He was admitted again, but he went into a state of shock 5 h after admission. The patient eventually went into cardiopulmonary arrest immediately after an episode of hematemesis. Abdominal CT revealed an enlarged stomach and duodenum, filled with high-density massive contents, proximal to the area adjacent to the abdominal aorta (Fig. 2d Asterisk, E Arrowhead). He died of hemorrhagic shock despite cardiopulmonary resuscitation.

## Discussion and conclusions

We encountered two cases of rapidly progressive infectious aortic disease among patients, exhibiting the characteristic emphysematous changes around the aorta. Due to its rapid clinical course, infectious aortic disease lesions rupture more frequently. Moreover, its mortality was reportedly high, compared to that of non-infectious aortic disease [3]. Therefore, early detection of this pathological entity is essential. However, in many cases, it is first recognized when the arterial wall is injured, and an aneurysm begins to appear, rather than a state of infectious arteritis immediately after an infection. Once the diagnosis is established, the administration of potent antibiotics should be initiated immediately based on the blood culture results.

According to the Mayo Clinic, the causative bacteria for this disease included gram-positive cocci in 50% of cases (30% *Staphylococci*, 20% *Streptococci*) and gram-negative bacilli in 35% (20% *Salmonella*, 15% *Escherichia coli*) [4]. In common, anaerobic bacteria or intestinal bacteria are famous as gas producing bacteria. *Klebsiella pneumoniae* is a gram-negative, facultative anaerobic, rod-shaped bacterium, whose serotype produces gas [5]. Most infected patients tend to develop complicated DM. There have been several cases of infectious aortic aneurysms secondary to *Klebsiella pneumoniae*, particularly from eastern Asia. However, mediastinal emphysema rarely occurs preceding aneurysmal formation [6]. In the first case, it is unknown whether the route of infection to the aortic wall is hematogenous or spread from the mediastinum. However, the fact remains that the patient had sepsis and the emphysematous changes were due to infection. The patient remained asymptomatic until day 25, when the aortic arch ruptured and showed pseudo aneurysmal formation. Chest CT on day 11 demonstrated no aneurysmal formation, but air pockets in the mediastinum were detected. Thereafter, the infectious aortitis adjacent to the mediastinal emphysema progressed and ruptured to form pseudo aneurysm.

Currently, contrast-enhanced CT is the preferred diagnostic imaging modality [7]. However, some patients have a negative blood culture result and nonspecific clinical manifestations. To address these situations, 18 F-fluoro-2-deoxy-D-glucose positron emission tomography/CT examination, a sensitive, metabolic, non-invasive imaging tool, is used to diagnose and follow up inflammation and infection in the vascular system. It can also be used to aid in the diagnosis of this disease [8]. This modality allows immediate assessment of the response to anti-inflammatory treatment and can guide optimal therapy. It must have been effective also in our two cases, but we could not apply it for our cases due to its unavailability in our hospital.

In our cases, the diagnosis of infectious arteritis was based on the CT findings of emphysematous changes around the aorta. However, the procrastinated follow-up timing with imaging modalities led toan aortic rupture resulting in pseudo aneurysmal formation in the first case and an arterial rupture to the duodenum in the second case. Based on these cases, initiating treatment for infectious aortic diseases earlier, conducting appropriate observation using imaging modalities such as CT, and cooperating with vascular surgeons improved the prognosis of patients. A previous report showed that hemoptysis was identified as a critical sign of an aortobronchial fistula [9]. It is vital to remain vigilant for this kind of red flag signs in the daily clinical settings.

Infectious aortic disease is a life-threatening condition that can be treated appropriately. Although rare, emphysematous changes are considered red flag signs during the early stage of infectious aortic disease, so we should be more vigilant for this kind of feature.

## Data Availability

Data sharing is not applicable to this article as no datasets were generated or analyzed during the current study.

## Abbreviations

CRP: C-reactive protein
CT: Computed tomography
DM: Diabetes mellitus
WBC: White blood cell
